# Litter mixture decomposition enhances the accumulation of soil active carbon and nitrogen in an alpine grassland

**DOI:** 10.3389/fmicb.2026.1801190

**Published:** 2026-03-18

**Authors:** Xiaogang Dong, Zhiyang Zhang, Zhangwen Liu, Yixuan Li, Jianjun Cao, Shiting Zhang

**Affiliations:** 1 State Key Laboratory of Herbage Improvement and Grassland Agro-ecosystems, College of Ecology, Lanzhou University, Lanzhou, China; 2 Key Laboratory of Resource Environment and Sustainable Development of Oasis of Gansu Province, College of Geography and Environmental Science, Northwest Normal University, Lanzhou, China; 3 Key Laboratory of Ecological Safety and Sustainable Development in Arid Lands, Northwest Institute of Eco-Environment and Resources, Chinese Academy of Sciences, Lanzhou, China

**Keywords:** alpine grassland, litter mixing effect, mixed litter decomposition, soil enzyme activity, soil microbial biomass

## Abstract

Litter is an important hinge connecting plants and soil, and its decomposition is a crucial process of nutrient cycling. However, the litter mixing effects (ME) on main functions related to soil carbon (C), nitrogen (N), and phosphorus (P) cycling, particularly in alpine ecosystems, remain unclear. Here, we incubated four single litters and six mixtures formed by pairwise combinations of single litter in the field for 630 days in an alpine grassland on the Tibetan Plateau, to determine ME (additive, synergistic or antagonistic effect) on soil functional indicators, including total and dissolved soil nutrients, microbial biomass, and enzyme activities. The results showed that: (1) Mixed litter decomposition produced mostly non-additive effects on soil indicators, of which synergistic effects were more prevalent than antagonistic effects. Specifically, soil dissolved organic carbon (SDOC), soil dissolved organic nitrogen (SDON), soil microbial biomass nitrogen (SMBN), soil β-1,4-glucosidase (BG) activity, and soil acid phosphatase (AP) activity exhibited mostly synergistic effects, accounting for 66.7%, 50.0%, 60.0%, 53.3%, and 40.0% of all cases, respectively. In contrast, only three indicators were dominated by additive effects, with 83.3% for soil organic carbon (SOC), 60.0% for soil total phosphorus (STP), and 56.7% for soil urease (URE) activity. (2) Strength of ME on soil functional indicators exhibited variability, with an average of 17.10% for SDOC, 18.65% for SDON, 28.74% for SMBN, 8.64% for BG activity, and 5.26% for AP activity due to synergistic effects. In contrast, there was an average decrease of 16.57% for STP due to antagonistic effects. (3) Strength of ME on SDOC, SDON, SMBN, and activities of BG and AP was correlated with cellulose (CE), P, and C/N ratio of litter. Our findings highlight litter mixtures enhance the accumulation of soil active C, N, and promote hydrolase activities in a synergistic way, and strengths of these effects were regulated by litter chemical traits. These findings suggest that the coexistence of plant species with contrasting litter chemical traits can accelerate the recovery of soil fertility through synergistic litter mixing effects in degraded alpine grasslands, thereby contributing to the functional maintenance of alpine grassland ecosystems.

## 1 Introduction

Plant litter is an important hinge connecting plants and soil, and its decomposition and nutrient return to soil are key processes in soil carbon (C), nitrogen (N), and phosphorus (P) cycling (Schmidt et al., 2011; Wieder et al., 2013; Jackson et al., 2017). The return of nutrients from litter to the soil affects key ecological processes, such as soil nutrient turnover, microorganisms assemble, and extracellular enzyme activities (Wagg et al., 2014; Gill et al., 2021). Litter in terrestrial ecosystems mostly exists in mixed species, and is likely to produce litter mixing effects (ME) on soil functional indicators during decomposition. These effects include non-additive effects (i.e., synergistic and antagonistic effect) and additive effect (Li et al., 2016; Qu et al., 2025). Understanding how this litter mixture affects soil functions is crucial because litter decomposition is the primary pathway for C and nutrient cycling.

Generally, the mixing effects of litter decomposition have primarily focused on decomposition rate, mass loss, and nutrient release dynamics (Gartner and Cardon, 2004; Hättenschwiler et al., 2005; Makkonen et al., 2013; Wang et al., 2014; Li et al., 2016; Mukamparirwa et al., 2024), but it is not clear whether these findings can be used to predict the mixing effects on soil indicators. The magnitudes of nutrient return to soil of each component within mixed litter vary with their chemical traits, and therefore the ME on soil physicochemical and biological properties is also different (Scott and Binkley, 1997; Mark et al., 2004; Buckeridge et al., 2010; Liu et al., 2022; Buchanan et al., 2024). Numerous studies have examined the effects of litter mixtures, revealing that non-additive effects account for a significant proportion of mass loss or nutrient dynamics (Gartner and Cardon, 2004; Hättenschwiler et al., 2005; Zeng et al., 2018), and non-additive effects are particularly likely to occur in heterogeneous mixtures, such as high-low nitrogen mixtures (Lecerf et al., 2011). However, the primary focus of these studies has remained on the decomposition of the litter itself, with limited research directly investigating the cascading ME on the underlying soil. A few studies have shown that the mixed litter decomposition produced synergistic, antagonistic or additive effects on indicators related to soil C, N, and P cycling, depending on decomposition stage and specific soil parameters (Heemsbergen et al., 2004; Gartner and Cardon, 2004; Jiang et al., 2013; Min et al., 2025). Consequently, it is generally considered that the effects of mixed litter decomposition on soil ecological processes remain poorly constrained. Moreover, the direction and strength of non-additive effect (synergistic and antagonistic effect) also varies with decomposition durations (Lee et al., 2014). Therefore, it is important to conduct mixed litter decomposition over a long period until mixed litter decomposes completely, to fully understand the mixing effects of litter decomposition on soil function.

The findings from the limited researches on soil responses to mixed litter are often inconsistent (Meier and Bowman, 2010). For instance, some studies showed that ME on soil organic matter, total N content, microbial biomass and enzyme activities were synergistic effects (Miao et al., 2019; Castro-Díez et al., 2019). In contrast, other studies reported that soil microbial biomass and enzyme activities showed antagonistic effects or additive effects (Chen et al., 2015a, 2017). Mixed litter decomposition increased soil dissolved organic nitrogen (SDON) by 4.5%, but decreased soil dissolved organic carbon (SDOC), microbial biomass carbon (SMBC) and urease (URE) activity by 7.7%, 3.4%, and 1.4%, respectively (Chen et al., 2017). SMBC and soil microbial biomass nitrogen (SMBN) were higher than those of single litter after mixed litter decomposition (Bai et al., 2021). These inconsistencies are likely due to the complex interactions among litter chemical traits (Meier and Bowman, 2008; Chen et al., 2015a). These contradictory results indicate the fragmented and incomplete understanding of how litter mixtures influence key soil functional indicators. Therefore, a systematic investigation integrating multiple decomposition stages and diverse soil parameters is essential to unravel the effects of mixed litter on soil nutrient cycling, particularly in alpine grassland ecosystems.

The Tibetan Plateau, often referred to as the third pole of the Earth, is the highest altitude and largest area on the earth (Yang et al., 2007), and alpine grasslands cover about two-thirds of the whole Tibetan Plateau (Zhou, 2001). Low temperatures in the region slow decomposition of organic matter, while the availability of N and P frequently limits soil fertility (Zhao and Zhou, 1999). Thus, litter decomposition plays a key role in regulating C and nutrient cycling in this area. Prior researches have examined how litter mixtures influences soil nutrient pools (Jiang et al., 2013; Chen et al., 2015b). However, there is a critical knowledge gap regarding ME on soil active C and N pools such as SMBC, SMBN, SDOC, and SDON, as well as enzyme activity, particularly under long-term *in situ* conditions. Here, we conducted a 630-day field incubation experiment using four contrasting leaf litters and their pairwise mixtures, by integrating multiple soil active C and N pools indicators and enzyme activity to identify likely effects on these soil functional indicators, and attempted: (1) to determine the ME (i.e., additive, synergistic, or antagonistic effect) on soil functional indicators; and (2) to elucidate the relationships between litter chemical traits and the ME on soil indicators.

## 2 Materials and methods

### 2.1 Study site

**Figure 1:**
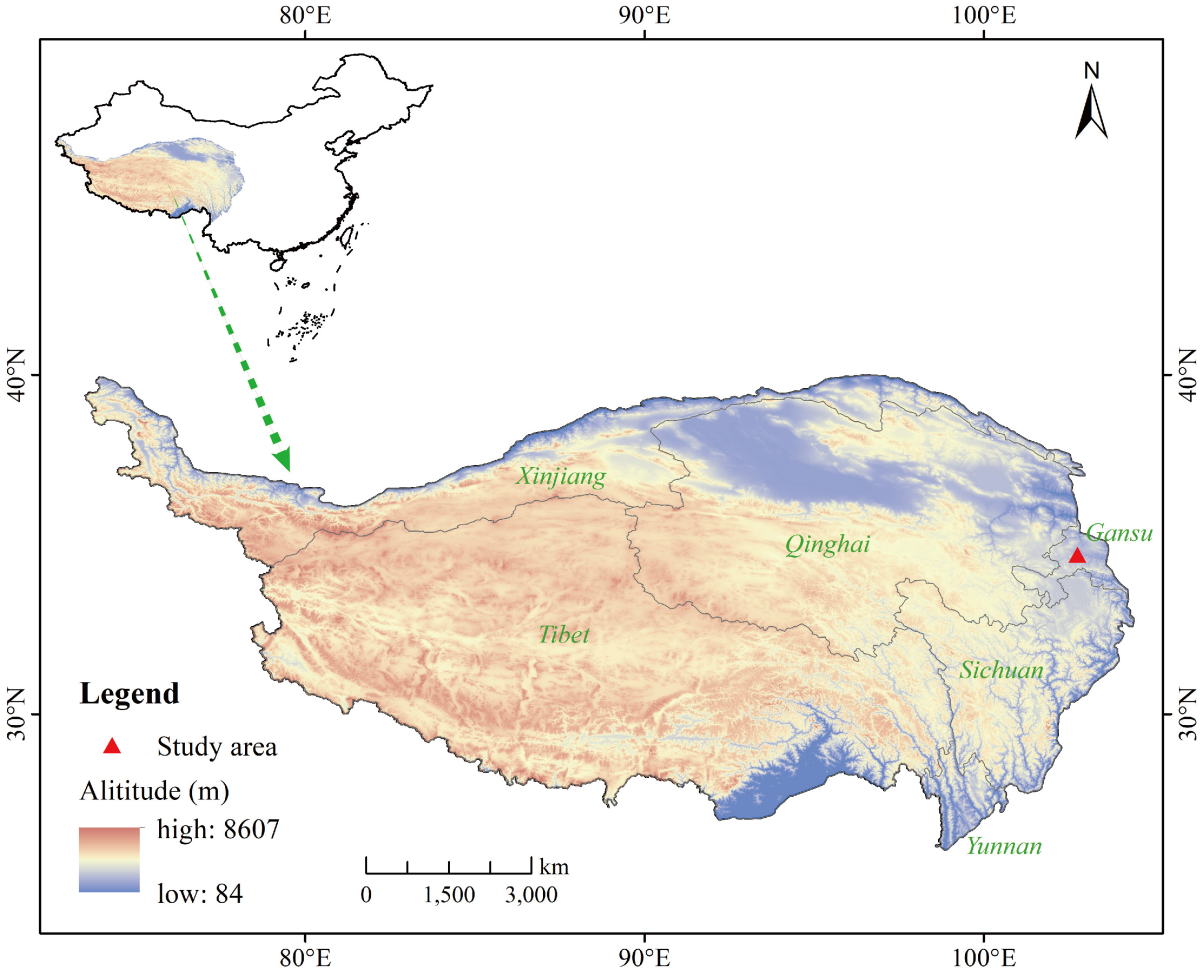
The study area.

This experiment was established at the Gannan Grassland Ecosystem National Observation and Research Station, located in Hezuo (N34°55', E102°53', 2,900 m a.s.l.) (Figure 1), Gansu Province, China. The mean annual temperature is 2.1 °C, the coldest months are December, January, and February, with an average temperature of −8.9 °C, and the warmest months are June, July, and August, with an average temperature of 11.5 °C. The mean annual precipitation is 570 mm, mainly occurring from June to August (growing seasons). Vegetation is alpine grassland, dominated by species including *Elymus nutans, Kobresia humilis, Leymus secalinus, Saussurea hieracioides, Gentiana straminea, Thermopsis lanceolate*, and *Ligularia virgaurea*. The soil type is classified as alpine meadow soil according to the Chinese Soil Classification System.

### 2.2 Experimental design

In this study, four common leaf litter species with contrasting quality, including *Thermopsis lanceolata* (*Tl*), *Gentiana straminea* (*Gs*), *Saussurea hieracioides* (*Sh*), and *Leymus secalinus* (*Ls*), were selected as experimental materials (Supplementary Table S1). We collected freshly senescent leaves in October 2017. The collected leaf litter was naturally air-dried at room temperature, and then cut into about 10 cm sections.

The litter decomposition was simulated using the litterbag method. The litterbags were constructed from 20 cm × 15 cm nylon nets with a mesh size of 2 mm × 2 mm to allow microorganisms and most soil fauna to enter the bags and contribute to the decomposition. This setup facilitated a realistic decomposition process driven by biotic interactions. Four plant litters were mixed in pairs to create six mixed litter treatments, so a total of 10 treatments including four single litter treatments (*Ls, Sh, Gs*, and *Tl*) and six mixed litter treatments (*Ls* × *Sh, Ls* × *Gs, Ls* × *Tl, Sh* × *Gs, Sh* × *Tl*, and *Tl* × *Gs*) were applied in this study. Each litterbag was filled with a standardized mass of 15 g for single treatments, while mixed litter treatments contained 7.5 g of two separate species.

This experiment lasted from 1st November 2017 to 24th July 2019, the experiment was a randomized block design with five replicate blocks, and the space between adjacent blocks was separated by 1 m wide buffers. Five incubation periods (180, 270, 360, 540, and 630 days after decomposition) were assigned within an incubation period. Fifty litter bags (10 treatments × 5 incubation periods) were randomly placed within each block and separated by 50 cm to prevent mutual interference. Therefore, 250 litter bags (10 treatments × 5 incubation periods × 5 replicates) were used in this study.

### 2.3 Sampling and measurements

At each sampling date, 10 litter bags were randomly collected in each block, three surface-soil cores (10 cm depth) underlying each selected litter bag were collected and then homogenized to a composite soil sample. After removing the impurities, the soil was filtered through a 2-mm mesh sieve. All fresh soil samples were divided into two subsamples. The first was refrigerated at 4 °C for the determination of soil dissolved organic carbon (SDOC), soil dissolved organic nitrogen (SDON), soil microbial biomass carbon (SMBC), soil microbial biomass nitrogen (SMBN), soil β-1,4-glucosidase (BG) activity, soil urease (URE) activity, and soil acid phosphatase (AP) activity, while the second was air-dried for the determination of soil organic carbon (SOC), soil total nitrogen (STN), soil total phosphorus (STP), soil available nitrogen (SAN), and soil available phosphorus (SAP).

SOC was determined by external heating with sulphuric acid-potassium dichromate, and STN and STP were determined using a fully automated intermittent chemical analyser (Smartchem 200 AMS/Westco, Italy). SAN was determined by the alkaline hydrolysis diffusion method, and SAP content was determined by NaHCO_3_ leach-molybdenum blue colourimetric method. Ten gram fresh soil was extracted with 0.5 M K_2_SO_4_ by shaking at 25 °C for 30 min and then centrifuged for 10 min (7,000 r/min). The supernatant was decanted and vacuum filtered through a 0.45 μm nitrocellulose membrane filter, and the filtrate was analyzed for SDOC and SDON using a TOC analyzer (Multi N/C 2100S, Analytikjena, Germany). SMBC and SMBN were determined by a chloroform fumigation-extraction method. The C and N content in extracts before and after fumigation were measured as described for SDOC and SDON above. Soil BG activity was quantified by the colorimetric method of nitrosalicylic acid, soil URE activity was determined by the colorimetric method of sodium phenol-sodium hypochlorite, and soil AP activity was assessed by the method of p-nitrobenzene disodium phosphate (Guan, 1986).

Lignin, cellulose, and hemicellulose of litter were analyzed using a Fibertec^TM^ 8000 Fiber Analyzer (Foss Technology, Hillerød, Denmark). Litter C, N, and P were also measured using the same methods as used for the soil.

### 2.4 Index calculation

The following formulas were used to calculate the litter mixing effects (ME) produced by mixed litter decomposition on the relevant indicators of soil C-, N-, and P-cycling (Butenschoen et al., 2014), as shown in Equations 1 and 2:


$$\begin{array}{lll} ME\left(\%\right) & = & \left(\frac{Observed_{AB}-Predicted_{AB}}{Predicted_{AB}}\right)\times 100 \end{array}$$ (1)

$$\begin{array}{lll} Predicted_{AB} & = & \left(Observed_{A}+Observed_{B}\right)/2 \end{array}$$ (2)

Where, *A* and *B* indicate the two components of mixed litter, “*Observed*_*AB*_” represents the measured concentration of soil indicator affected by the mixed litters with species litter *A* and *B*, while “*Predicted*_*AB*_” denotes the value which was calculated using the measured concentration of soil indicator affected by the two single species litter *A* and *B*, respectively. The average strength of *ME* for a given soil indicator was calculated using all *ME* data. The *ME* deviates from 0 (*P* < 0.05), indicating that the ME on soil indicators was non-additive. That is, a value of *ME* > 0 indicates that the ME on soil indicators presented a synergistic effect, while a value of *ME* < 0 indicates that the ME on soil indicators presented an antagonistic effect. If there was no discernible difference between *ME* and 0 (*P* > 0.05), the ME on soil indicators exhibited an additive effect.

### 2.5 Data analysis

All experimental data were expressed as mean ± standard error (SE). Descriptive statistics were employed for all data. Assumptions of normality (Shapiro-Wilk test) and homogeneity of variance (Levene's test) were verified prior to analysis. Paired *t*-tests were used to test whether additive, synergistic and antagonistic effects on soil functional indicators of litter mixtures significantly differed from zero. Redundancy analysis (RDA) was performed to examine the relationships between litter chemical traits and ME on soil indicators. Prior to RDA, multicollinearity among litter chemical traits was assessed using variance inflation factors (VIF), and variables with VIF > 10 were removed. All the statistical analyses were conducted using SPSS 22.0 (SPSS Inc., Chicago, USA) with a significance level of *P* < 0.05.

## 3 Results

### 3.1 ME on soil organic carbon and nutrients

**Figure 2:**
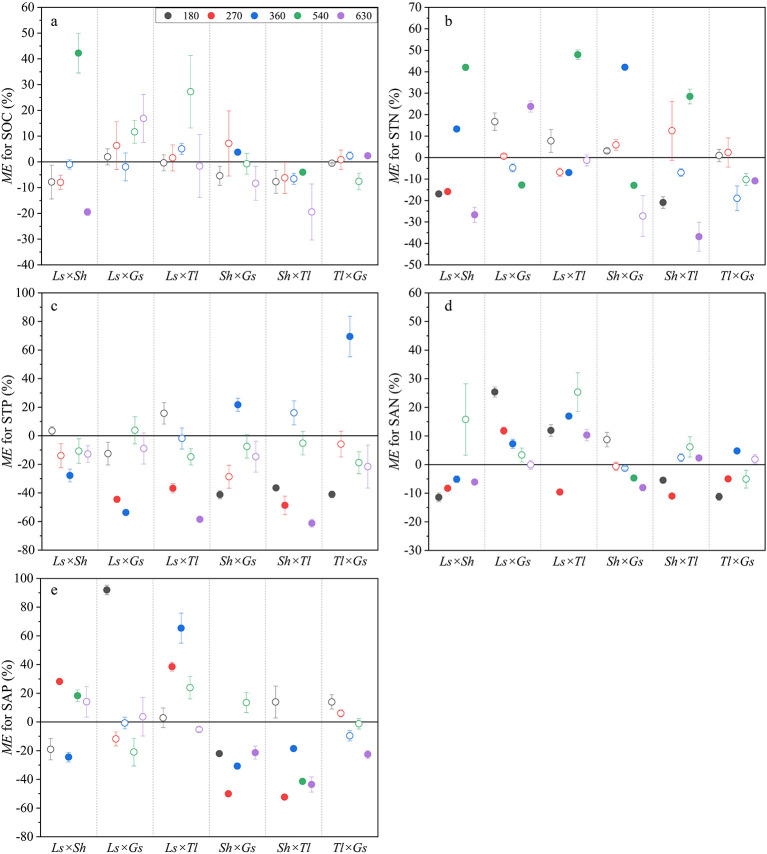
Litter mixing effects (ME) on SOC **(a)**, STN **(b)**, STP **(c)**, SAN **(d)**, and SAP **(e)**. *ME* values relative to the y = 0 line. The solid symbols represent statistically significant non-additive effects (*P* < 0.05), *ME* > 0 indicates a synergistic effect, while *ME* < 0 indicates an antagonistic effect. The hollow symbols represent additive effects that are not significantly different from 0 (*P* > 0.05).

Across litter mixtures, SOC and STP mostly exhibited additive effects, accounting for 83.3% and 60.0% of all cases, respectively (Figures 2a, c). The non-additive effect of mixed litter decomposition on SOC was found after 630 days for *Ls* × *Sh* and *Tl* × *Gs* (Figure 2a). The number of non-additive and additive effects on STN were largely equal, and *Ls* × *Sh* consistently demonstrated non-additive effects, with antagonistic effects being the most prevalent (Figure 2b). A total of 33.3% and 6.7% of mixed litters exhibited antagonistic and synergistic effects on STP, respectively (Figure 2c). Mixed litters exhibited non-additive effects on SAN in 63.3% of all cases, of which 36.7% demonstrated antagonistic effects. Meanwhile, *Ls* × *Sh* mainly exhibited antagonistic effects while *Ls* × *Gs* and *Ls* × *Tl* demonstrated synergistic effects (Figure 2d). Non-additive effects on SAP accounted for 50.0% of all cases, of which 33.3% showed antagonistic effects, for instance, *Sh* × *Gs* and *Sh* × *Tl* mainly exhibited antagonistic effects (Figure 2e).

### 3.2 ME on soil dissolved carbon and dissolved nitrogen

**Figure 3:**
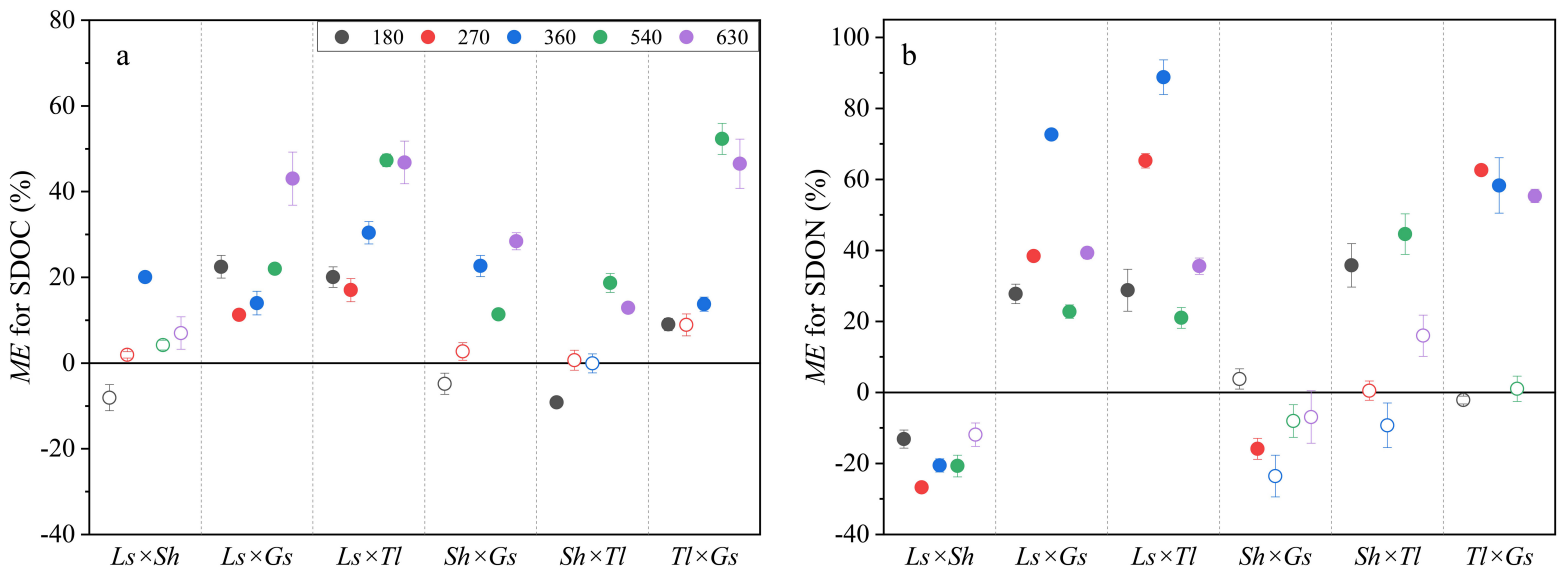
Litter mixing effects (ME) on SDOC **(a)** and SDON **(b)**. *ME* values relative to the y = 0 line. The solid symbols represent statistically significant non-additive effects (*P* < 0.05), *ME* > 0 indicates a synergistic effect, while *ME* < 0 indicates an antagonistic effect. The hollow symbols represent additive effects that are not significantly different from 0 (*P* > 0.05).

Mixed litter decomposition mainly produced non-additive effects on SDOC and SDON, with the proportion of 70.0% and 66.7% of all cases, respectively. Synergistic effects showed 66.7% and 50.0% for SDOC and SDON across litter mixtures, respectively, indicating synergistic effects were more prevalent than antagonistic effects (Figures 3a, b). For SDOC, only *Sh* × *Tl* produced an antagonistic effect after 180 days, while *Ls* × *Gs* and *Ls* × *Tl* completely exhibited synergistic effects in the whole decomposition duration (Figure 3a). For SDON, *Ls* × *Sh* mainly demonstrated antagonistic effects, while *Ls* × *Gs, Ls* × *Tl*, and *Tl* × *Gs* mainly exhibited synergistic effects (Figure 3b).

### 3.3 ME on soil microbial biomass and enzyme activities

**Figure 4:**
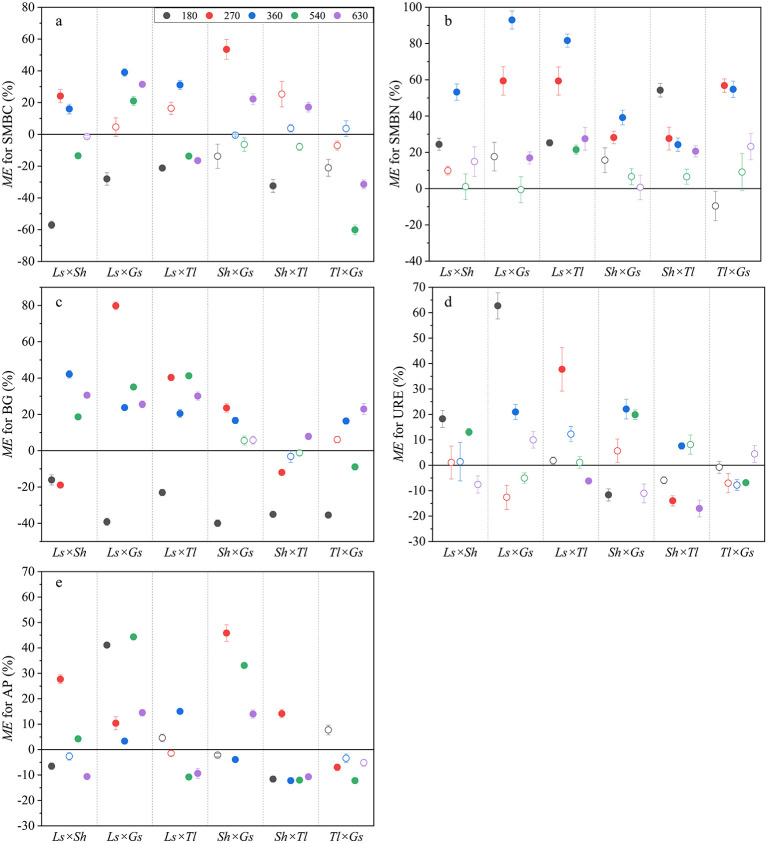
Litter mixing effects (ME) on SMBC **(a)**, SMBN **(b)**, BG activity **(c)**, URE activity **(d)**, and AP activity **(e)**. *ME* values relative to the y = 0 line. The solid symbols represent statistically significant non-additive effects (*P* < 0.05), *ME* > 0 indicates a synergistic effect, while *ME* < 0 indicates an antagonistic effect. The hollow symbols represent additive effects that are not significantly different from 0 (*P* > 0.05).

SMBC and SMBN demonstrated non-additive effects, with the proportion of 60.0% and 60.0% of all cases, respectively (Figures 4a, b). With regard to SMBC, 30.0% of mixed litters exhibited antagonistic effects (Figure 4a). With regard to SMBN, no antagonistic effect was observed, and all mixed litters with the exception of *Ls* × *Tl* exhibited additive effects after 540 days (Figure 4b). For soil BG activity, the additive, synergistic, and antagonistic effects were found to account for 16.7%, 53.3%, and 30.0% of all cases, respectively (Figure 4c). With regard to the non-additive effects, all mixed litters demonstrated antagonistic effects on BG activity after 180 days. Subsequently, most mixed litters exhibited synergistic effects (Figure 4c). For soil URE activity, the additive, synergistic, and antagonistic effects accounted for 56.7%, 26.7%, and 16.6% of all cases, respectively (Figure 4d). For soil AP activity, the additive, synergistic, and antagonistic effects accounted for 23.3%, 40.0%, and 36.7% of all cases, respectively (Figure 4e). During the majority of decomposition duration, *Ls* × *Gs* and *Sh* × *Gs* demonstrated synergistic effects, while *Sh* × *Tl* exhibited antagonistic effects, and *Tl* × *Gs* exhibited additive effects (Figure 4e).

### 3.4 The strength of ME on soil functional indicators

**Figure 5:**
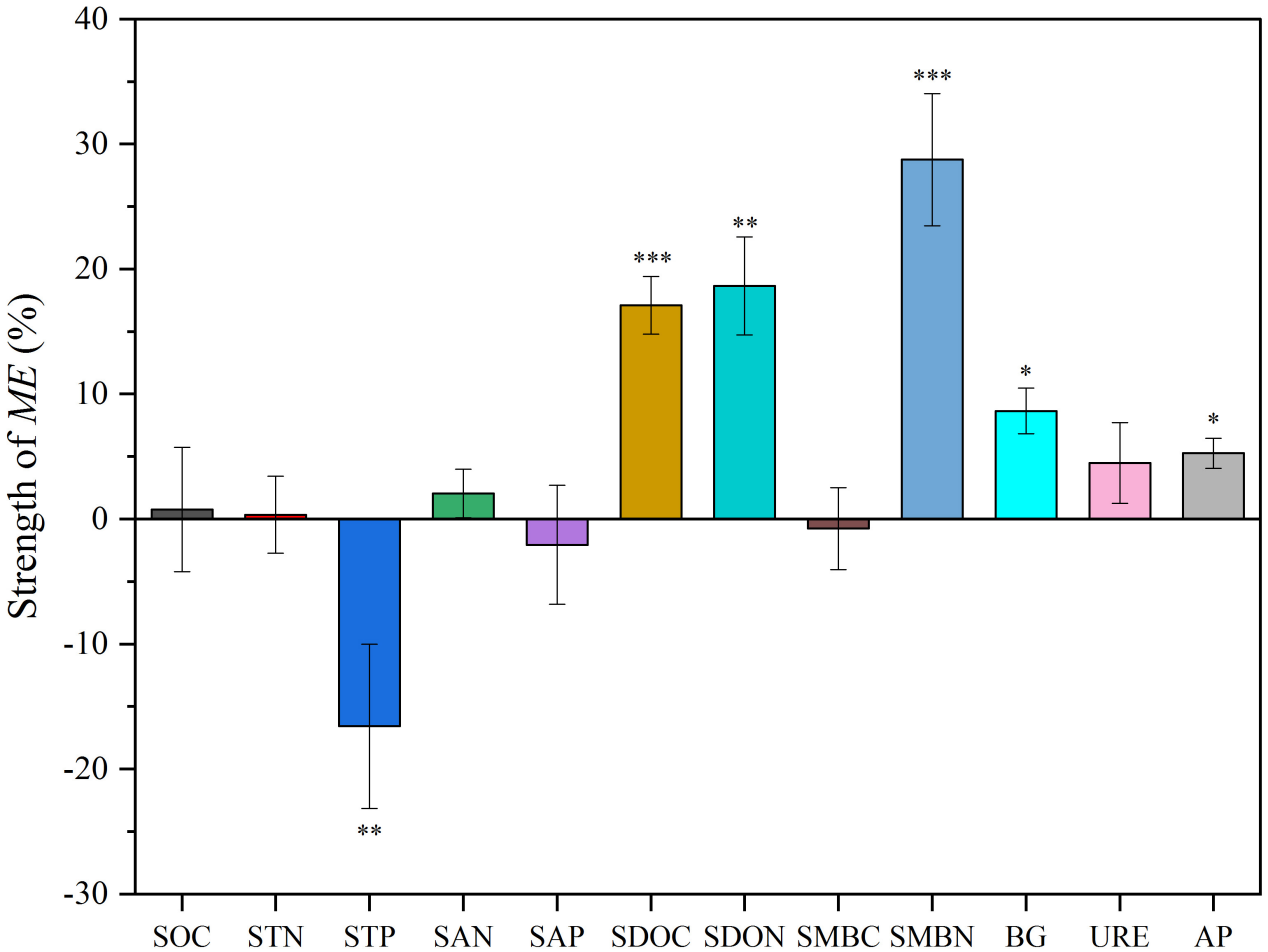
The average strength of litter mixing effects (ME) on soil functional indicators during the incubation period. Asterisks denote significant differences tested against zero: ***P < 0.001, **P < 0.01, *P < 0.05.

Within 630 days of decomposition, the average strength of ME produced by the six mixed litters on the measured soil functional indicators was different (Figure 5). The decomposition of mixed litters resulted in an increase in SOC (0.76%), STN (0.34%), SAN (2.04%), SDOC (17.10%, *P* < 0.001), SDON (18.65%, *P* < 0.01), SMBN (28.74%, *P* < 0.001), BG activity (8.64%, *P* < 0.05), URE activity (4.47%), and AP activity (5.26%, *P* < 0.05) in the topsoil. In particular, SDOC, SDON, and SMBN exhibited the most pronounced increase. However, STP (−16.57%, *P* < 0.01), SAP (−2.06%), and SMBC (−0.77%) in the topsoil were reduced by mixed litter decomposition.

### 3.5 Relationships between litter chemical traits and ME on soil indicators

**Figure 6:**
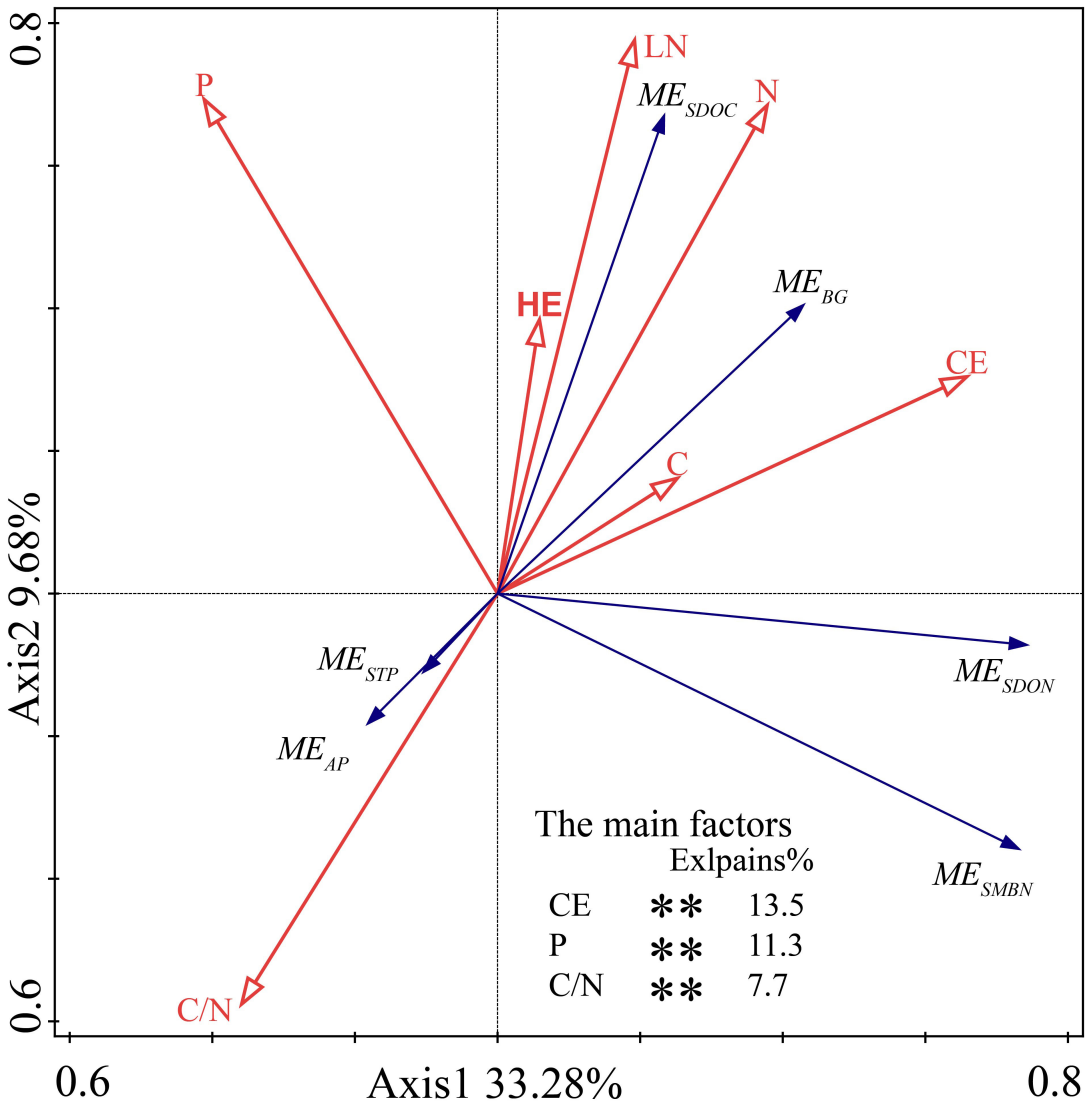
Redundancy analysis (RDA) of the dominant drivers for litter mixing effects (ME) on soil functional indicators during the incubation period. C, Litter carbon; N, Litter nitrogen; P, Litter phosphorus; C/N, Litter C/N ratio; LN, Litter lignin; CE, Litter cellulose; HE, Litter hemicellulose. **P < 0.01.

RDA showed that mixed litter chemical traits explained 42.96% of the total variation in ME on soil functional indicators, with the first two axes accounting for 33.28% and 9.68% of the variance, respectively. CE, P and C/N of mixed litter were identified as the most significant drivers of ME on soil indicators (Figure 6). ME on SDOC and BG activity were positively correlated with litter CE, while negatively correlated with litter C/N. ME on STP and AP activity were positively correlated with litter C/N, while negatively correlated with litter CE. ME on SDON was positively correlated with litter CE, while negatively correlated with litter C/N and P. ME on SMBN was negatively correlated with litter C/N and P.

## 4 Discussion

### 4.1 Mixing effects of litter decomposition on soil C and nutrients

This study indicated that SOC and STP exhibited mostly additive effects, in contrast, STN showed approximately equal additive and non-additive effects (Figures 2a–c). Meanwhile, the ME observed in SOC and STN were minor, whereas those in STP were large (Figure 5). Soil total C, N, and P pools integrate slower stabilization pathways (e.g., microbial necromass formation, aggregation and mineral association), leading to predominantly additive responses to litter mixtures (Huang et al., 2004; Hu et al., 2006; Zhu et al., 2024). Although the decomposition of mixed litter introduces C sources into the soil, it also enhances soil respiration (Chen et al., 2017; Zhang et al., 2024). This results in the decomposition of SOC and the release of carbon dioxide (CO_2_) into the atmosphere, thus reducing soil carbon sequestration (Guo et al., 2021). Specifically, the accelerated microbial decomposition of both C in the mixed litter input and native C may have offset the expected carbon accumulation (Sokol et al., 2019). Consequently, the effects of mixed litter on the quantity and quality of SOC are limited (Hollesen et al., 2015), confirmed by this study. Although mixed litter produced mostly additive effects on STP (Figure 2c, accounting for 60.0% of all cases), it generally reduced STP over the entire decomposition period (Figure 5). This may be attributed to more antagonistic effects than synergistic effects (e.g., antagonistic effects accounted for 33.3% of all cases, while synergistic effects accounted for 6.7% of all cases) (Figure 2c). Additionally, our study showed that there was a positive relationship between ME on STP and litter C/N (Figure 6), indicating that a lower litter C/N could enhance the antagonistic effects on STP (Pang et al., 2021). This was confirmed by the finding that mixed litters incorporating *Gs* or *Tl* (both with low C/N) had antagonistic effects on STP (Figure 2c). In this study, antagonistic effects were observed more frequently than synergistic effects on SAN. This finding suggests that litter mixture is more likely to inhibit soil N availability. Specifically, in N-limited alpine ecosystems, litter with relatively low N content contributed minimally to soil N availability during decomposition (Jiang et al., 2013), as confirmed by the observation that the *Ls* × *Sh* mixture (where both *Ls* and *Sh* have low N content) induced antagonistic effects on SAN (Figure 2d). The strength of ME on SAP was −2.06% (Figure 5), due to the inhibition of soil P availability during the late stage of litter decomposition (Figure 2e). As decomposition proceeds, increased microbial activity and biomass can enhance microbial P immobilization, temporarily reducing the pool of available P in soil (Bünemann et al., 2012; Qualls and Richardson, 2000).

Litter mixtures exerted synergistic effects on soil active pools such as SDOC and SDON (Figure 3), and strength of ME was 17.10%, and 18.65%, respectively (Figure 5), indicating that litter mixing primarily accelerated C and N cycling processes over the study period. These findings were consistent with the results of previous studies in different ecosystems (Kalbitz and Kaiser, 2008; Jiang et al., 2013; Chen et al., 2015a). The present study found that both ME on SDOC and SDON were negatively correlated with litter C/N (Figure 6), indicating that high litter quality (low C/N) tended to induce synergistic effects for SDOC and SDON. This relationship can be explained by two mechanisms: firstly, high-quality litter (low C/N) can alleviate microbial nutrient limitation, enhance microbial metabolism and promote the production and exudation of extracellular enzymes (Allison and Vitousek, 2005), which in turn accelerate the breakdown of complex organic matter into soil soluble forms (e.g., SDOC and SDON). For example, litter mixtures incorporating *Gs* or *Tl* (both with low C/N) resulted in synergistic effects on SDOC and SDON (Figure 3). Secondly, soil dissolved organic matter (SDOM) is closely associated with the leaching of surface-mixed litter (Wang et al., 2021). When litter-leaching enters the soil at an early decomposition stage, it rapidly prompts active soil C and N (Zhu et al., 2024). The diverse chemical composition of mixed litter can enhance the leaching of soluble compounds, as well as the synthesis and deposition of SDOM (Dang et al., 2025), thereby rendering synergistic effects discernible in these rapidly responding fractions. Furthermore, SDOM is an immediately available substrate for soil microorganisms, responding rapidly to litter leachates and microbial metabolic products to amplify detectable litter mixing effects (Strid et al., 2016; Hensgens et al., 2021; Li et al., 2021).

In the Tibetan Plateau, low temperatures constrain decomposition, but seasonal moisture variability and freeze–thaw cycles can enhance litter fragmentation and pulse-like leaching, which may lead to an increase in SDOC and SDON driven by litter mixtures (Wang et al., 2012; Jiang et al., 2021). Therefore, even if litter mixing does not cause a rapid increase in SOC and STN, it can substantially elevate active C and N fractions that regulate microbial activity and nutrient availability. Overall, our findings suggest litter mixing can accelerate active organic matter turnover in this alpine grassland.

### 4.2 Mixing effects of litter decomposition on soil microbial biomass

Our findings showed that ME on SMBC was predominantly non-additive (accounting for 60.0%), with a comparable proportion of synergistic and antagonistic effects (Figure 4a), and totally weak (Figure 5). Our results diverged from other studies in which most mixed litters exhibited pronounced synergistic effects on SMBC (Jiang et al., 2013; Chen et al., 2017). This discrepancy may be attributed to composition and decomposition duration of litter mixtures, both of which are well known to affect soil microbial biomass through mixing effects (Chen et al., 2020; Ai et al., 2023). In this study, the minor ME on SMBC likely resulted from increased microbial activity and respiration constrained net microbial carbon accumulation. Furthermore, SMBC is one of the most responsive indicators of SOC changes and tends to fluctuate in parallel with SOC (Díaz-Raviña et al., 1993). We also found the decomposition of mixed litter produced less additive effects and more synergistic effects on SMBN across all of incubation times and litter mixtures (Figure 4b), which was consistent with the results of previous studies in alpine meadows (Meier and Bowman, 2010). Additionally, the results showed litter mixtures involving low-quality (high C/N) *Ls* combined with high-quality (low C/N) *Tl* or *Gs* frequently produced synergistic effects on SMBN. The synergistic effects on SMBN can be mechanistically explained by stoichiometric complementarity of soil microbes due to inputs of heterogeneous substrates into soil (Meier and Bowman, 2010; Wang et al., 2024). In this study, the selected single litter exhibited significant and contrasting C, N, and P stoichiometry (Supplementary Table S1), and we found that the ME on SMBN was negatively correlated with litter C/N and P (Figure 6). This indicated that rapidly decomposing litter with low C/N ratio supplies soil microorganisms with diverse nutrients in varying quantities, and thus have a higher probability of triggering non-additive effects in microbial biomass. Specifically, litter mixtures with contrasting chemical traits provide diverse C and N sources, alleviating microbial nutrient imbalances and promoting the more efficient utilization of organic substrates by microbes. This, in turn, enhances microbial N immobilization and biomass N accumulation, resulting in positive non-additive responses in SMBN (Meier and Bowman, 2008). Additionally, the negative relationship between SBMN and litter P in this study suggests that P may decrease the strength of synergistic response to litter mixture for SBMN. Furthermore, this study found that SMBN increased significantly after litter mixing, likely due to a substantial rise in SDOC and SDON (Figure 3), which are regarded as potential sources of C and N for microbial growth (Kalbitz et al., 2000).

### 4.3 Mixing effects of litter decomposition on soil enzyme activities

In this study, soil BG and AP activities showed relatively high proportions of positive non-additive responses to litter mixture, whereas URE activity showed mainly additive responses to litter mixture (Figures 4c–e), indicating that ME was differential for C-, N-, and P- acquiring enzymes. Furthermore, ME on BG activity displayed pronounced temporal shifts, with early antagonism followed by later synergy (Figure 4c), highlighting that mixing effects on BG activity are decomposition stage-dependent. Similarly, some studies also found that the non-additive effects on soil BG activity was significantly associated with the decomposition stage (Mao et al., 2025). Additionally, our results showed that mixed litter decomposition increased the soil enzyme activities involved in C and P cycles (e.g., BG and AP activities) (Figure 5), indicating that mixed litter produces more available substrates and nutrients for soil microbial growth and reproduction. The present study demonstrated a positive correlation between ME on BG activity and litter CE (Figure 6), suggesting litter CE can enhance soil microbial investment in C-acquiring enzymes. In alpine soils where P-limitation is common (Bing et al., 2016), it has been demonstrated that soil microorganisms require more soil P for growth during litter decomposition, thereby stimulating the synthesis of soil AP (Freedman et al., 2016; Martins et al., 2021). Our results showed that ME on AP activity was positively correlated with litter C/N (Figure 6), indicating high C/N in litter mixtures facilitated synergistic effects on soil AP activity (Zeng et al., 2021). The synergic effects on soil enzyme activities may result from the increase in active soil organic matter (e.g., SDOC and SDON) following mixed litter decomposition in this study, which was confirmed as the most important factors affecting soil enzyme activities in previous studies (Gmach et al., 2020; Błońska et al., 2021; Wang et al., 2024).

## 5 Conclusions

The decomposition of mixed litter mainly produced non-additive effects on functional indicators of soil C, N, and P cycling, with the synergistic and antagonistic effects occurring alternately, and the synergistic effects were more prevalent than the antagonistic effects. These synergistic effects resulted in an increase in SMBN, SDOC, SDON, and the activities of soil BG and AP. Furthermore, the strength of these synergistic effects was regulated by litter chemical traits. These results indicated that the decomposition of mixed litter was more conducive to the accumulation of soil active C and N, and to the promotion of hydrolase activities. Our findings suggest that multiple plant species community can promote soil functions through mixed litter decomposition in alpine grassland ecosystems.

## Data Availability

The original contributions presented in the study are included in the article/Supplementary material, further inquiries can be directed to the corresponding author.
