# Supplementary material for: Litter mixture decomposition enhances the accumulation of soil active carbon and nitrogen in an alpine grassland
Source: Front Microbiol. 2026 Mar 18;17:1801190. doi: 10.3389/fmicb.2026.1801190 (PMC13038913; doi:10.3389/fmicb.2026.1801190)
Supplement: Supplementary file 1 [file Table_1.docx]

Supplementary Material

**Table S1.** Initial chemical traits of four single litters.

| Variables | *Ls* | *Sh* | *Gs* | *Tl* |
| --- | --- | --- | --- | --- |
| C (g/kg) | 504.17±8.07 a | 421.49±6.20 bc | 443.37±4.19 b | 410.76±5.05 c |
| N (g/kg) | 8.52±0.12 c | 8.95±0.17 c | 10.52±0.19 b | 18.64±0.14 a |
| P (g/kg) | 1.25±0.03 b | 1.04±0.03 c | 1.44±0.02 a | 0.88±0.01 d |
| LN (%) | 8.08±0.07 b | 7.04±0.13 c | 6.91±0.16 c | 9.11±0.19 a |
| CE (%) | 38.49±0.93 a | 14.58±0.51 c | 14.72±0.70 c | 17.29±0.81 b |
| HE (%) | 32.54±0.64 a | 10.10±0.58 c | 11.00±0.79 c | 15.43±0.91 b |
| C/N | 59.21±1.10 a | 47.15±1.06 b | 42.20±0.87 c | 22.05±0.44 d |
| LN/N | 9.49±0.05 a | 7.87±0.03 b | 6.57±0.04 c | 4.89±0.06 d |

Notes: Values are presented as mean ± standard error (SE). At the end of the field arrangement, for the four single litters, natural air-dried litters were accurately weighed at 15 g, with five replicates per litter, respectively. A total of twenty samples from four single litters were used to determine the initial chemical traits. *Ls*: *Leymus secalinus*; *Sh*: *Saussurea hieracioides*; *Gs*: *Gentiana stramine*; *Tl*: *Thermopsis lanceolate*; C: Litter carbon; N: Litter nitrogen; P: Litter phosphorus; LN: Litter lignin; CE: Litter cellulose; HE: Litter hemicellulose. Different letters in the same line indicated significant difference among litters (*P* < 0.05).
